# Probable Transmission of SARS-CoV-2 from African Lion to Zoo Employees, Indiana, USA, 2021

**DOI:** 10.3201/eid2906.230150

**Published:** 2023-06

**Authors:** Audrey A. Siegrist, Kira L. Richardson, Ria R. Ghai, Brian Pope, Jamie Yeadon, Betsy Culp, Casey Barton Behravesh, Lixia Liu, Jennifer A. Brown, Leslie V. Boyer

**Affiliations:** Potawatomi Zoo, South Bend, Indiana, USA (A.A. Siegrist, B. Culp);; Indiana Department of Health, Indianapolis, Indiana, USA (K.L. Richardson, B. Pope, J. Yeadon, L. Liu, J.A. Brown);; Centers for Disease Control and Prevention, Atlanta, Georgia, USA (R.R. Ghai, C. Barton Behravesh);; University of Arizona, Tucson, Arizona, USA (L.V. Boyer)

**Keywords:** COVID-19, coronavirus disease, SARS-CoV-2, African lion, severe acute respiratory syndrome coronavirus 2, viruses, respiratory infections, zoonoses, vaccine-preventable diseases, rapid test, RT-PCR, risk factor, biosecurity, zoonoses, One Health, Indiana, United States

## Abstract

We describe animal-to-human transmission of SARS-CoV-2 in a zoo setting in Indiana, USA. A vaccinated African lion with physical limitations requiring hand feeding tested positive for SARS-CoV-2 after onset of respiratory signs. Zoo employees were screened, monitored prospectively for onset of symptoms, then rescreened as indicated; results were confirmed by using reverse transcription PCR and whole-genome virus sequencing when possible. Traceback investigation narrowed the source of infection to 1 of 6 persons. Three exposed employees subsequently had onset of symptoms, 2 with viral genomes identical to the lion’s. Forward contact tracing investigation confirmed probable lion-to-human transmission. Close contact with large cats is a risk factor for bidirectional zoonotic SARS-CoV-2 transmission that should be considered when occupational health and biosecurity practices at zoos are designed and implemented. SARS-CoV-2 rapid testing and detection methods for big cats and other susceptible animals should be developed and validated to enable timely implementation of One Health investigations.

Although SARS-CoV-2 is primarily transmitted from person-to-person (*1*), it is considered zoonotic because of natural infections observed in a range of mammalian species (*2*,*3*). The broad host range of SARS-CoV-2 is evident from natural infections occurring in zoos, sanctuaries, and aquaria, most frequently in big cats (*3*), but also in mustelids (*4*), nonhuman primates (*5*), and others. Zoo outbreaks typically begin after close contact with an infected zookeeper (*6*–*10*). One Health investigations have shown transmission of SARS-CoV-2 from human to animals (*4*,*8*,*9*,*11*–*14*), but examples of animal-to-human transmission are rare (*15*–*19*).

We report a multispecies cluster of SARS-CoV-2 infections associated with an infected African lion (*Panthera leo*) at a seasonal, mid-sized, Association of Zoos and Aquariums–accredited zoo in Indiana, USA. The lion’s likely source of exposure was a fully vaccinated, asymptomatic employee, and forward transmission from the lion to other fully vaccinated employees probably occurred.

## Methods

### Setting 

The sentinel case occurred in December 2021, when the zoo was closed for the season. The lion was housed alone in a building with an indoor/outdoor enclosure located >30 feet from other animal enclosures. Feeding and veterinary procedures were conducted indoors by dedicated staff with assigned key access. Susceptible species elsewhere in the zoo included a snow leopard (*Panthera uncia*), Amur tigers (*Panthera tigris altaica*), Amur leopards (*Panthera pardus orientalis*), North American river otters (*Lontra canadensis*), and several species of nonhuman primates. All susceptible animals, including the lion, had received 2 doses of Zoetis experimental mink coronavirus vaccine (https://www.zoetis.com) during September–November 2021. Animal health was overseen by a full-time onsite veterinarian.

Zoo practice before the outbreak included SARS-CoV-2 prevention measures from the Zoo and Aquarium All Hazards Partnership (*20*,*21*), such as suspending behind-the-scenes tours and close-distance behavioral training for susceptible species. Employees were required to complete the COVID-19 vaccination series, practice social distancing, monitor for COVID-19 symptoms, and, when ill, exclude themselves from work. Disinfectant foot baths were placed at enclosure entrances, and high-pressure cleaning methods were replaced by methods less likely to aerosolize infectious material. Employees were required to wear surgical masks at all times and nitrile gloves during feedings.

### Sentinel Case

The lion was a geriatric (20-year-old) male with chronic renal insufficiency and severe degenerative intervertebral disc disease prohibiting normal range of motion for self-grooming of the caudal body. He was vaccinated with the Zoetis vaccine on September 14 and October 7, 2021. Routine care included hand feeding twice daily, therapeutic laser treatments of the spine and hindquarters, corticosteroids, gabapentin, renal supplements, and mesenchymal stem cell treatments. On December 18, employees observed coughing, dyspnea, shivering, lethargy, sneezing, nasal discharge, anorexia, and exposed nictitans. Marbofloxacin treatment was initiated. Nasal swabs were collected on December 18 and 23. He was euthanized on December 23 because of declining mobility associated with intervertebral disc disease. The acute clinical signs attributed to SARS-CoV-2 had largely resolved and were not a factor in the euthanasia decision.

### Investigation

**Figure 1:**
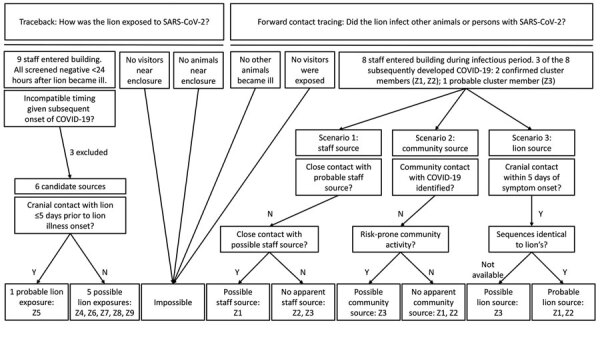
Traceback and forward contact tracing investigations of SARS-CoV-2 transmission between an African lion and zoo employees, Indiana, USA, 2021–2022. The traceback investigation narrowed the potential source of the lion’s SARS-CoV-2 infection to 6 zoo employees who had lion contact within 10 days, 1 of whom (employee Z5) had cranial contact within 5 days of the lion’s illness onset but did not have close contact with employees Z1, Z2, or Z3. Possible human sources were identified for Z1 (close occupational contact with Z7) and for Z3 (community activity), although in neither case were these potential sources shown to carry the virus. Employees Z1, Z2, and Z3 all had symptoms and had confirmed SARS-CoV-2 infection 3 days after their most recent cranial contact with the sick lion.

We initiated an investigation on December 18 to identify the source of the lion’s exposure and the potential for forward transmission (Figure 1). We defined a confirmed cluster-associated case as illness in a human or animal at the zoo with laboratory evidence for the same strain as the sentinel case, based on genomic sequencing of a clinical sample. We defined a probable cluster-associated case as illness in a human or animal at the zoo with laboratory evidence for SARS-CoV-2 based on reverse transcription PCR (RT-PCR) or rapid antigen test of a clinical sample but for which no genomic sequencing information was available.

For both traceback and forward contact tracing investigations, we assumed the exposure period to begin 10 days before illness onset, with probable acquisition within 5 days prior. We assumed the infectious period to begin 2 days before illness and extend 10 days after illness onset. We considered any person who entered the enclosure during the lion’s exposure or infectious period to be a lion contact. We classified the care provided by lion contacts as cranial (e.g., feeding and nasal swabbing with <2 feet between human and lion heads) or caudal (e.g., arthritis care and injections performed ≈9 feet from the lion’s head). We defined close contact between persons as proximity of <6 feet for >15 minutes in 1 day.

#### Sentinel Case Investigation

We screened nasal swab samples from the lion onsite on December 18 by using a lateral flow immunoassay validated for human use (DiaTrust COVID-19 Ag Home Test; Celltrion, https://www.celltrionhealthcare.com). We sent aliquots of samples to the US Department of Agriculture’s National Veterinary Services Laboratories for confirmatory testing. Necropsy was performed on December 23 at the Michigan State University Veterinary Diagnostic Laboratory.

#### Traceback Investigation

We screened nasopharyngeal swab samples from all employees exposed to the lion within the 10 days before his illness onset onsite by using lateral flow immunoassay on December 18 and 19. We reviewed personnel schedules, keeper and veterinary daily reports, maintenance logs, lion treatment schedules, veterinary examination notes, security logs, and social media pages for dates and nature of interactions among lion contacts.

#### Forward Contact Tracing Investigation

We screened lion contacts with subsequent symptoms suggestive of infection onsite for SARS-COV-2 by lateral flow immunoassay within 24 hours of symptom onset. The Indiana Department of Health confirmed the results by using a TaqPath COVID-19 Combo Kit RT-PCR (ThermoFisher, https://www.thermofisher.com).

An Indiana Department of Health epidemiologist conducted interviews of infected personnel. Those interviews covered symptoms, vaccination history, and exposures to other zoo employees and the public during the 10 days before their onset of illness. We verified location history for activity outside the zoo for these persons through credit or debit card transaction data where possible. We cross-referenced contact data with zoo records. We deidentified data summaries for analysis.

Relatedness of genomic sequences was analyzed by the authors (L.L., B.P., and J.L.) by comparing samples with 10 closely related samples collected during August 2021–February 2022 throughout the state of Indiana. We sequenced all samples in 1 run by using Clear Dx WGS SARS-CoV-2 Reagent Kit 2.0 (Clear Labs, https://www.clearlabs.com), which contained midnight primers.

We used the Pangolin COVID-19 Lineage Assigner (https://pangolin.cog-uk.io) to identify lineage from FASTA files generated by Clear Dx. We constructed the phylogenetic tree in CLC Genomics Workbench 21.0.3 (QIAGEN, https://www.qiagen.com) using classical sequencing analysis tools.

### Infection Control

Upon detection of SARS-CoV-2 in the lion, employees providing care for susceptible species at the zoo were required to wear N95 respirators. Those entering the lion building also used face shields, disposable gowns, shoe covers, and gloves. Lion building access was restricted further, and laser treatments were discontinued. Work assignments were altered so that employees providing care to the lion would not come in contact with other susceptible animals (*22*).

## Results

### Sentinel Case Investigation

We detected evidence of SARS-CoV-2 in the lion through onsite screening on December 18. We detected further evidence of SARS-CoV-2 by RT-PCR in nasal swab samples from December 18 and December 23, and in nasal turbinate, lung tissue, and intestinal tissues obtained at necropsy on December 23. Extracted RNA from both nasal swab samples produced high-quality 29,707-nt sequences for genomic sequencing (SARS-CoV-2 Delta variant, AY.103 lineage). Necropsy confirmed intervertebral disc degeneration, chronic renal disease, chronic lower airway disease, and severe rhinitis.

### Traceback Investigation

**Figure 2:**
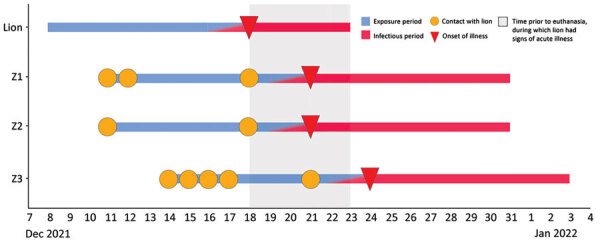
Timeline of probable transmission of SARS-CoV-2 from an African lion to zoo employees, Indiana, USA, 2021–2022. The lion’s likely exposure period was December 8–17, and his infectious period was December 16 through euthanasia on December 23. During the lion’s infectious period, employees Z1 and Z2 each had a single day of cranial contact with him, coinciding with the day of the lion’s illness onset. Z3 had cranial contact with the lion on 3 occasions during his infectious period. Figure highlights the lack of overlap between Z1 and Z2’s infectious period and the lion’s exposure period, and between Z3′s infectious period and Z1 and Z2’s exposure periods.

Nine people (employees Z1–Z9) entered the lion building at some time during the lion’s potential exposure period during December 8–18 (Figure 1). All 9 reported being asymptomatic on December 18. Eight had screening tests on December 18, and 1 had a screening test on December 19; all were negative. Employees Z1, Z2, and Z3 had onset of COVID-19 during December 21–24, with infectious periods that began on or after December 19 (Figure 2). Employee Z5 had cranial contact during the lion’s 5-day probable acquisition period. Employees Z4, Z6, Z7, Z8, and Z9 had lion contact 6–10 days before onset of the lion’s illness, performed caudal activities exclusively, or both.

### Forward Contact Tracing Investigation

No additional animals at the zoo had illness suggestive of SARS-CoV-2 infection (Figure 1). Eight persons entered the lion building during the lion’s infectious period (December 16–23). Employees Z1, Z2, and Z3 were fully vaccinated and previously healthy, each of whom was involved in cranial care 3 days before having onset of upper respiratory symptoms. Each of those 3 employees was positive on rapid antigen test within 24 hours after symptom onset and had subsequent RT-PCR confirmation of SARS-CoV-2 infection. Samples from patients Z1 and Z2 were suitable for sequencing; the sample from Z3 was of insufficient quality for sequencing.

**Figure 3:**
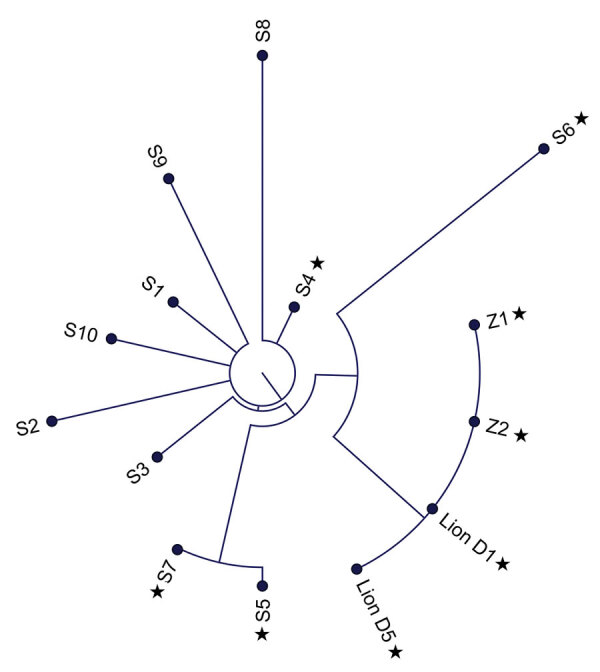
Phylogenetic tree of SARS-CoV-2 Delta variant, AY.103 lineage, genome sequences from an African lion (day 1 and day 5) and 2 zoo employees (Z1 and Z2) shown in comparison with reference sequences from COVID-19 patients from 7 counties in Indiana, USA, August 2021–February 2022. Reference sequences are labeled chronologically as S1 to S10. Stars indicate specimens collected in December 2021.

The sequences generated from lion samples on December 18 and 23 were identical to those from Z1 and Z2 samples. The 10 community comparators varied from the cluster sequences by 12–24 nt (Figure 3).

Employees Z5, Z8, and Z9 had no close contact with Z1, Z2, or Z3 during their exposure periods. Z4 had close contact with Z2 on December 18, the day that both had negative screening tests. Z6 entered the lion enclosure at the same time as Z3, but was maintaining >6 feet distance, 9 days before Z3’s symptom onset. Z7 had close contact with Z1 on December 11, 12, and 18. Z7 worked in the lion enclosure with Z2, but that contact took place a full 10 days before Z2’s onset of symptoms and was at >6 feet for <15 minutes. Z7 also worked in the lion enclosure with Z3 for <15 minutes and at a distance of >6 feet 8 days before Z3’s onset of symptoms. Employee Z3 had no known exposures outside of the zoo but did participate in community social activities with risk for unrecognized transmission of SARS-CoV-2 on several occasions during the 10 days before onset of symptoms.

## Discussion

This multispecies cluster of SARS-CoV-2 included 3 confirmed cases (2 human, 1 felid) and 1 probable case (human). The identical genomic sequences detected in samples from the confirmed cases demonstrate that the infections were acquired in a common setting. Given that the zoo was closed to visitors, the source of infection for the sentinel case was almost certainly 1 of 6 asymptomatic employees who tested negative on the day of the lion’s diagnosis and who subsequently reported no signs of illness. Among those 6, employee Z5, the only person who had cranial contact within 5 days before the lion’s onset of illness and who did not later have symptomatic COVID-19, was the most likely source of the lion’s infection.

To determine whether lion-to-human transmission took place, we considered 3 scenarios (Figure 1). The first scenario posits that zoo employees acquired infection from the same human source as the lion. Although employee-to-employee transmission could not be ruled out on an individual basis, no pathway of transmission could be identified that explained all probable cases within the cluster.

The second scenario posits that zoo employees acquired infection from an unrelated community source. For employee Z3, whose viral genome was unknown, community acquisition is possible. Employees Z1 and Z2 had low-risk social behavior with no other identified sources of SARS-CoV-2 transmission from the community, and samples from both yielded sequences genetically identical to the lion’s viral strain. Although it is technically feasible that each of them, independently, could have acquired the cluster strain from asymptomatic community carriers, the chance of this happening are extremely low.

The third scenario posits that at least 1 zoo employee acquired infection from the lion. During cranial-end procedures, the lion breathed, roared, and coughed within arm’s length of staff. Each person that had symptom onset after the lion was involved in cranial-end care while the lion was sick, and each had symptoms 3 days after that exposure. Lion-to-human transmission of SARS-CoV-2 is, therefore, the most probable explanation for their infections.

Our investigation strongly suggests that lion-to-human transmission took place in 2, and possibly 3, instances, which is important for at least 2 reasons. First, animal-to-person transmission of SARS-CoV-2 is an occupational health risk for veterinary and animal care staff that interact closely with susceptible animals. Second, transmission occurred despite an up-to-date SARS-CoV-2 vaccination history in every person involved, including the person or persons who likely transmitted the virus to the lion, the lion, and the person or persons who likely became infected from the lion. Although SARS-CoV-2 transmission from vaccinated persons to vaccinated zoo animals has been documented previously (*23*), our results show that transmission can also occur from vaccinated zoo animals to vaccinated persons. Many human vaccine efficacy studies indicate reductions in COVID-19 disease severity after vaccination (*24*,*25*); however, further research is needed to determine if the same is true of zoonotic SARS-CoV-2 transmission among vaccinated persons and animals.

The unique setting for these cases minimized the number of potential exposure pathways required to reach this conclusion. Initiation of the investigation and heightened safety measures on the day of sentinel case discovery improved analysis and contained spread. Timely screening, prospective monitoring for symptom development, cross-referencing of occupational and interview data, and genomic sequencing combined to allow inferences of causality.

Swabbing of leonine nasal passages for SARS-CoV-2 diagnosis has been reported (*26*), but neither the sampling method nor the use of tests designed for human diagnosis has been formally validated. Rapid testing methods applicable to animals should be developed and validated to enable timely implementation of biosecurity measures.

The first limitation of our study is the limited number of community samples that were available for the sequencing analysis. Second, rapid tests may not reliably establish SARS-CoV-2 negative status. Third, the distance associated with close contact and exposure and infectious periods for lions were assumed to be similar to those for humans, although these have not been established. Finally, diminished immune response of this geriatric, chronically ill lion may have precluded a robust response to vaccination, limiting the generalizability of conclusions related to viral shedding after vaccination.

Timely human-animal-environmental assessments and implementation of appropriate biosafety interventions are essential for both animal and human health during community outbreaks of SARS-CoV-2. A One Health approach, which connects the health of persons, animals, and environment (*27*), should be used to respond to susceptible zoo animals, particularly those requiring close human contact, that develop clinical signs compatible with SARS-CoV-2 infection. Close contact with large cats should be considered a risk factor for bidirectional zoonotic SARS-CoV-2 transmission, regardless of prior immunization. This consideration may be especially warranted in the cases of geriatric animals or those with underlying health conditions.
